# Assessment of the Isoniazid Preventive Therapy Uptake and Associated Characteristics: A Cross-Sectional Study

**DOI:** 10.1155/2018/8690714

**Published:** 2018-05-06

**Authors:** Francine Mwayuma Birungi, Stephen Graham, Jeannine Uwimana, Brian van Wyk

**Affiliations:** ^1^Department of Epidemiology and Biostatistics, School of Public Health of the College of Medicine and Health Sciences, University of Rwanda, Kigali, Rwanda; ^2^Faculty of Community and Health Sciences, University of Western Cape, Cape Town, South Africa; ^3^Centre for International Child Health, University of Melbourne Department of Paediatrics and Murdoch Children's, Research Institute, Royal Children's Hospital, Melbourne, VIC, Australia; ^4^International Union against Tuberculosis and Disease, Paris, France

## Abstract

**Objective:**

To assess the uptake of isoniazid preventive therapy (IPT) by eligible children in Kigali, Rwanda, and associated individual, households, and healthcare systems characteristics.

**Methods:**

A cross-sectional study was conducted among child contacts of index cases having sputum smear-positive pulmonary tuberculosis. Data were collected from 13 selected primary health centres. Descriptive statistics were used to generate frequency tables and figures. Logistic regression models were performed to determine characteristics associated with IPT uptake.

**Results:**

Of 270 children (under 15 years), who were household contacts of 136 index cases, 94 (35%) children were less than 5 years old and eligible for IPT; and 84 (89%, 95% CI 81–94) were initiated on IPT. The reasons for not initiating IPT in the remaining 10 children were parents/caregivers' lack of information on the need for IPT, refusal to give IPT to their children, and poor quality services offered at health centres. Factors associated with no uptake of IPT included children older than 3 years, unfriendly healthcare providers, HIV infected index cases, and the index case not being the child's parent.

**Conclusion:**

The National Tuberculosis Program's policy on IPT delivery was effectively implemented. Future interventions should find strategies to manage factors associated with IPT uptake.

## 1. Introduction

In 2015, the World Health Organisation (WHO) estimates showed that 10% of the 9 million tuberculosis (TB) incident cases occurred in children, which resulted in 210,000 TB-related deaths including 170,000 in HIV-negative children [1]. Children exposed to index cases with TB, particularly sputum smear-positive pulmonary TB (PTB), are at risk of infection and when infected, infants and young children (<5 years old) are at high risk of developing the disease [2, 3]. WHO recommends routine screening of child contacts in resource-limited settings through a symptom-based screening approach that can be implemented in the community and provision of preventive therapy for at-risk contacts after excluding TB [4]. The most widely recommended regimen is isoniazid preventive therapy (IPT) that is provided as a daily dose for at least 6 months. Notwithstanding the potential benefits of contact screening for active case detection and initiation of IPT, these activities are rarely implemented in TB endemic settings [5, 6]. Furthermore, even when IPT is offered to eligible children, its uptake is often poor [7, 8].

Rwanda is a TB endemic resource-limited country, which had an estimated 6.6 [95% IC: 5.6–7.6] thousand new TB cases in 2015 [1]. According to the TB surveillance system, 5,763 TB cases, including 68.1% of new confirmed bacteriological pulmonary TB cases and 85.3% of sputum smear-positive PTB cases, were reported in the period between 2015 and 2016. Children under 15 years old represented 5.3% of all TB cases reported [9]. The active contact screening and IPT are recommended by the National TB Programme (NTP) in Rwanda but TB case detection strategy is limited to passive screening. Although guidelines for IPT have been in existence since 2005, their implementation has not been assessed. For a few years, particular attention has been given to TB in children by Rwanda's NTP since TB treatment is recognized as an opportunity that prevents and addresses an important cause of child mortality [10, 11]. The NTP strategy has promoted the uptake and adherence of IPT as one of the 30 performance indicators since 2009. This paper reports about the uptake of IPT by eligible children in Kigali, the capital city of Rwanda, and evaluates the associated individual, households, and healthcare systems characteristics.

## 2. Methodology

### 2.1. Site Selection

A cross-sectional study was conducted at 13 primary health centres (PHCs) providing TB diagnostic and treatment services in Kigali. Kigali, the capital city of Rwanda, reports the highest prevalence of TB in Rwanda and around 30% of Rwanda's total pulmonary TB (PTB) cases [9]. Thus, Kigali was selected as case study site. There are 35 primary health centres (PHCs) in Kigali whence 23 PHCs provide TB diagnostic and treatment services and represent entry points for TB cases. The criterion used to select 13 PHCs from the 23 PHCs providing TB diagnostic and treatment services was a record of at least 10 sputum smear-positive PTB cases reported between January and June 2015. Among the 13 PHCs selected for this study, nine (69%) were public-funded and four were faith-based (public and private funded). Of the 13 PHCs, three had two staff members, and ten (77%) had one staff member, working in TB services. All the staff members were trained in TB management and provided counseling to parents/caregivers on IPT before their children started the regimen. In Rwanda, medication for TB is provided free of charge. Moreover, all TB index cases are offered the opportunity to choose the nearest healthcare facility or community health worker they wish to receive TB treatment or IPT.

### 2.2. Study Population

This study was conducted among household contacts of index cases with sputum smear-positive PTB in 13 selected PHCs from 1 August 2015 to 29 February 2016. The criteria for selecting an index case of any gender were as follows: having at least one child under the age of 5 years, not belonging to a household having a previously selected index case, and providing proof of living in Kigali during the period of study. Identified index cases were requested, through either phone conversations or trained community health workers (CHWs), to bring their children to the nearest PHC on a specific day that coincided with data enumerators' visits to the PHC. In case the index cases were not parents/caregivers of the children needed at the PHCs, they were requested to inform those children's parents/caregivers to do so.

Eligible child contacts were aged below 5 years old and shared the same household with a selected index case within 3 months prior to the diagnosis of the latter. The children were enrolled following the signing of written informed consent by parents or primary caregivers. Ineligible child contacts included those born after the index cases were diagnosed and initiated on TB treatment, child contacts on TB treatment, and those that were not living in the same household with the index cases before the diagnosis. Moreover, child contacts older than 5 years, including those infected with HIV, were also excluded in accordance with Rwanda's NTP policy.

### 2.3. Data Collection and Management

Data were collected in twofold, from patients' records at the PHC facilities and from parents/caregivers of eligible child contacts interviewed by trained enumerators using a structured questionnaire. The questionnaire was developed, pretested during a pilot study in two selected sites, and modified for later use in data collection. Twelve selected data enumerators were trained to conduct interviews with parents/caregivers of selected child contacts. Additionally, 20 CHWs were trained to identify and enumerate all eligible children in the households as well as explain the study to parents/caregivers and sensitize them to take child contacts to the nearest PHCs for clinical evaluation and data collection. Furthermore, by screening each child and interviewing each parent/caregiver, data enumerators ensured that the child contact was eligible or otherwise excluded even if he/she has been declared eligible by CHWs.

The uptake of IPT is defined as the proportion of children eligible to receive IPT according to the WHO recommendation [12]. Screenings conducted during this study identified all children as eligible for IPT according to the WHO recommendation [12]. To assess the uptake of IPT, every parent/caregiver of an eligible child was asked whether the child was initiated on the IPT or not. The IPT register at PHCs assisted to verify the information given by parents/caregivers. The sociodemographic characteristics and medical history of index cases, such as a result of smear microscopy, HIV status, residential address, and telephone number, were recorded. The data collected were validated by the index cases, parents, or caregivers of selected children once they were identified to ensure their accuracy. All children underwent a history, physical, and chest X-ray (CXR) examination. Anteroposterior and lateral CXR were also performed on all children; they were read by two independent experienced general practitioners, trained in interpreting CXR and blinded to the clinical details of participants; an experienced radiologist verified all CXR to rule out any discordance. The components of all reports were agreed on before a final diagnosis was determined. Symptomatic children are treated with antibiotics for seven days as recommended by the current TB diagnostic algorithm in the country. Child contacts with persistent TB-related symptoms or abnormal CXR were referred to the District Hospital for further tests including smear microscopy, Xpert MTB/RIF assay, and solid culture using sputum collected through gastric lavage following the standard procedure [13] in order to exclude TB disease.

The interview with eligible children's parents/caregivers identified the sociodemographic and economic characteristics of the index cases, their households, and knowledge on how to prevent TB in child contacts. A parent/caregiver was considered knowledgeable about, first, IPT prevention if he/she knew that administering INH for 6 months would protect the child contacts against TB. Second, prevention of child contacts from contracting TB if he/she knew three of the following pieces of information: using a mask when breastfeeding, avoiding to kiss him/her, avoiding to sleep in the same room or bed with him/her, opening windows and doors for good ventilation, and using arm protection when coughing. The interview with eligible children's parents/caregivers also determined characteristics of the healthcare facility such as quality of services provided and the attitude of health providers towards patients. Those characteristics were assessed by asking parents/caregivers the level of satisfaction with the quality of services offered at the PHCs and whether the healthcare service providers were friendly.

### 2.4. Data Analysis

The data collected were double-entered into a Microsoft Excel worksheet and exported to Stata Software for statistical analysis after checking their consistency. The data were analyzed using descriptive and multivariate statistics. Continuous variables were dichotomized using the median as the cut-off. Categorical variables were described using frequency tables and proportionate methods. Univariate and multivariate logistic regression was performed to determine characteristics associated with IPT uptake. Where appropriate, Chi-square test or Fisher's exact test was performed to assess the association between two variables. Variables with a *p* value < 0.2 in univariate analysis were included in the logistical regression model using backward stepwise method. The final model included those factors that retained statistical significance. The odd ratios (OR) and adjusted OR (aOR) along with its 95% confidence interval were calculated using Stata Software (version 13). The associations were declared significant if *p* value < 0.05.

### 2.5. Ethical Approval

The Biomedical Research Ethics Committee of the University of the Western Cape and the Ethics Review Board of the University of Rwanda, College of Medicine and Health Sciences, approved the study protocol. Permission was obtained from Rwanda NTP to collect data from the eligible PHCs.

## 3. Results

**Figure 1:**
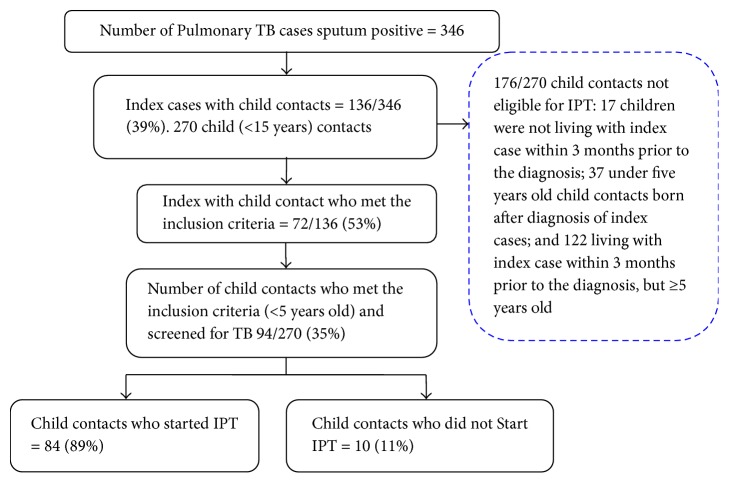
Flow of recruitment of child TB contacts.

Figure 1 shows the flow of recruitment of child contacts with 136 (39%) of 346 sputum smear-positive PTB index cases diagnosed and treated at 13 PHCs in Kigali during the 7-month study period. The index cases had at least one child contact aged between 0 and 14 years. Of the 136 index cases, 72 (53%) had at least a child contact who met the inclusion criteria. Of 94 (35%) children from 72 index cases who were eligible, 84 (89%) had started IPT.

Tables 1, 2, and 3 show the characteristics of the eligible index cases, their households, and child contacts by IPT uptake, respectively. The results show no significant difference between children who started IPT and those who did not with regard to the characteristics of index cases (Table 1) and healthcare facilities (that are unfriendly versus friendly and satisfaction level of parents/caregivers on the quality of services offered). There were significant differences between children who started IPT and those who did not with regard to the sex of the head of the household (13/62 versus 5/10, *p* = 0.049) (Table 2) and the age of the child contact (≤3 versus >3 years, *p* = 0.007) (Table 3). Children living in households headed by female were more likely not to be initiated on IPT than those living in households headed by a male. Also, children aged > 3 years old were more likely not to be initiated on IPT than those ≤ 3 years old. Tuberculosis-related symptoms such as coughing, fever, and weight loss were reported by 25% (23/94) of child contacts, and those cases reported responded to generic antibiotics recommended by the current TB diagnostic algorithm in the country. Neither of the children screened presented with an abnormal CXR nor a diagnosed TB disease [12, 14]. The majority of parents/caregivers of child contacts (66/72, 92%) had knowledge of at least one method on how to prevent transmission of TB to children and 32% (23/72) had knowledge of IPT prevention (Table 2).

All the 94 eligible child contacts were screened for TB by PHC providers whence 84 (89%, 95% CI 81–94) were initiated on IPT for 6 months as per the national and WHO guidelines [12, 14]. The reasons given by parents/caregivers for not initiating child contacts on IPT were lack of awareness of the need to do so (5/10 or 50%); failure to initiate IPT (4 or 40%); and poor healthcare service experienced at the PHC (10%).

In univariate analysis, the age of child contacts, sex of the household head, and relationship between healthcare providers and parents/caregivers were associated with the uptake of IPT (Table 4). Child contacts over 3 years old were more likely not to be initiated on IPT than those who were below 3 years old (OR 7, 95% CI 1.65–29; *p* < 0.008). Children living in households headed by a female were more likely not to be initiated on IPT than those living in households headed by a male (OR 4.6, 95% CI 1.18–17.9; *p* < 0.028). Child contact whose parents/caregivers did not find friendly healthcare providers at the PHCs were also more likely not to be initiated on IPT than those whose parents/caregivers did. (OR 10, 95% CI 1.26–83; *p* < 0.029).

In multivariate analysis, the sex of households' head had no significance. The final explanatory variables of no uptake of IPT were age group of child contacts (≤3 years versus >3 years), HIV status of child contacts (HIV-positive versus HIV-negative), relationship between child contacts and index cases (child versus others), HIV status of index case (HIV-positive versus HIV-negative), household income (income < 50.000 Rwandan Franc versus ≥ 50.000 Rwandan Franc), and relationship between healthcare providers and parents/caregivers (friendly versus unfriendly). After adjusting the variables, the age of child contacts and relationship between healthcare providers and parents/caregivers remained associated with the uptake of IPT. Child contacts older than 3 years were more likely not to be initiated on IPT than those less than 3 years old (aOR 29, 95% CI 2.17–400; *p* < 0.011). Moreover, the child contacts whose parents/caregivers found healthcare providers unfriendly were also more likely not to be initiated on IPT than those whose parents/caregivers found them friendly (aOR 19, 95% CI 2.51–140; *p* < 0.017). The HIV status of index cases and the relationship between child contacts and index cases were also associated with no uptake of IPT in multivariate analysis. Child contacts living with HIV-positive index cases were more likely not to be initiated on IPT than those living with HIV-negative ones (aOR 8.1, 95% CI 2.53–537; *p* < 0.038). Furthermore, the child contacts who were not children of index cases were more likely not to be initiated on IPT than those who were index cases' children (aOR 59, 95% CI 2.74–127; *p* < 0.009).

## 4. Discussion

The primary aim of this study was to assess the uptake of IPT by child contacts and associated factors in order to inform the NTP on its implementation. Despite the diversity methodology designs, the IPT uptake established in this study (89%) was found to be higher than 6% [15], 18.7% [16], 26.8% [17], 33% [18], and 64.3% [19] reported in Malawi, Timor-Leste, South Africa, South India, and Ethiopia, respectively. In contrast, recent studies conducted in the Gambia [20] and Benin [21] have reported an 89% and 99% of IPT uptake, which is similar to and higher than this study's findings, respectively. The integration of IPT into the programmatic delivery of healthcare might explain the high uptake reported in this study as well as in study findings reported in Gambia and Benin. This is in contrast to earlier studies that were conducted in a healthcare facility environment [15, 16]. Rwanda's NTP strategy adopts the households' visit of index cases by healthcare providers at the beginning of sputum smear PTB treatment. These visits allow for screening child contacts and initiating them on IPT. Our high IPT uptake finding corroborates results published in the 2013-2014 and 2015-2016 annual reports of Rwanda's NTP revealing an uptake of 85% and 78%, respectively. However, these reports do not provide information on the actual number of eligible contacts who had access to IPT in the community.

The WHO provided the first estimates of preventive therapy coverage for eligible children under the age of 5 years in 2016 [1]. The estimates showed that only 5.6% of an estimated 440,000 child contacts received preventive therapy in 2015 in the African region. However, Rwanda was among a few countries in Africa that provided data to the WHO from routine surveillance of preventive therapy for young child contacts [1]. Therefore, the high uptake in our study may reflect the particular attention being given by Rwanda NTP to TB in children in accordance with the Rwanda government's priority intervention aimed at preventing and addressing the most important causes of child mortality [10, 11]. Additionally, the goal of Rwanda NTP strategies is to strengthen more than 30 indicators outlined in the Performance-Based Financing (PBF) since 2009. The outlined indicators include “number of children eligible for IPT who received it” and “number of children aged below 5 years old who completed IPT.” The funding of healthcare services according to the PBF is based on the performance of medical facilities in enhancing the quantity and quality of preventive and curative treatment to the people [22]. Hence, the PBF has improved the quantity and quality of healthcare in many countries [23–25].

Our study showed that most parents/caregivers of child contacts had some general knowledge on how to prevent TB in children. That level of knowledge was higher than that reported in studies conducted elsewhere [26, 27]. Information, Education, and Communication sessions are organized twice a week at healthcare facilities by service providers, at the community level by the CHWs and at local politico-administrative authorities level as well as in media, to inform the population about TB. Other studies have shown that low-level knowledge on TB could negatively affect the health-seeking behaviour of the people [27, 28]. Contrasting findings have been reported in a study conducted in Malawi, where only 8% of parents with sputum smear-positive TB took their children to a medical clinic for screening despite having clear information on the need to do so [8].

The geographical accessibility can also explain the high uptake of IPT in Rwanda. Across the country, there has been an improvement in the ease of access to healthcare centres. Countrywide, the average time to access the nearest healthcare centre is less than 1 hour [29]. Transport cost was not a limiting factor mentioned by any parent/caregiver whose children were not initiated on IPT in our study as was the case in the study conducted in Malawi [30]. Furthermore, a study carried out in Bangkok, Thailand, by Tornee et al. [31] shows that the short distance from home to the nearest medical clinic was associated with adherence of the households' contact to screening.

In the univariate analysis, the significance of the sex of household heads was lost when it was adjusted for other variables. This finding suggests that the sex of index cases was a confounder variable in this study. Nevertheless, additional studies are needed to investigate the role of gender in the decision to initiate IPT.

In multivariate analysis, our study established that the child contacts who were not children of index cases were more likely not to be initiated on IPT than those who were their children. This finding corroborates a study conducted in Timor-Leste [16] as well as a qualitative study in Bangkok, Thailand [31]. Both studies reported lack of screening of child contacts who were not children of index cases. The approach is slightly different in Rwanda, whereby household visits of index cases with sputum smear-positive PTB by healthcare providers helps in screening child contacts and initiating them on IPT. Thus, child contacts who are not children of the index cases have a high possibility of being screened even though the initiation of IPT among these children may be low. When an index case is not the biological parent of a child contact, the latter may choose to be visited by a healthcare provider in the absence of the former. Often, healthcare providers inform the index cases about the intended home visit so that children can be screened but not necessarily start on IPT. This is because index cases may not inform the parents or caregivers of the children the need for initiating their children on IPT. This could explain the 100% screening of eligible child contacts in this study whence only 89% were initiated on IPT. The parents/caregivers of 50% of child contacts who were not initiated on IPT reported their lack of information about its usefulness.

Our study also established that child contacts older than 3 years were more likely not to be initiated on IPT than those aged below 3 years old. These findings can be explained by the fact that parents/caregivers protect their younger children from contracting TB more than their older children. Similar findings were reported in a qualitative study conducted in Bangkok, Thailand, by Tornee et al. [31], which showed that parents/caregivers worry that their younger children might get TB infection and take them to healthcare facilities for screening.

The parents/caregivers who found unfriendly healthcare providers at the PHCs were also more likely not to initiate their children on IPT than those who found them friendly. Child contacts living with HIV-positive index cases were less likely to be initiated on IPT than those living with HIV-negative index cases. Those two factors may be correlated. A study demonstrated that interactions and negative experience of people seeking treatment in government healthcare facilities contribute to a reduction in subsequent medical visits or follow-ups [32], which is mostly observed among the HIV-positive population.

Besides the negative experience from unfriendly healthcare providers, the HIV-positive people have to cope with social stigma [33, 34]. A study revealed that women, who often take children to healthcare facilities, experience stigma-related problems more than men [35]. In our study, 71% index cases were followed up in public PHCs and 56% of them whose children were not started on IPT were HIV infected. This suggests that negative experience in government healthcare facilities and social stigma among TB-HIV coinfected persons have a negative impact on the uptake of IPT among child contacts. This finding is a cause of concern in Rwanda since the healthcare-seeking behaviour of HIV-positive parents/caregivers influence TB screening and management in children.

The study has some limitations. First, the research was conducted in Kigali; thus, the findings might not be generalized to the whole country, especially remote rural areas where healthcare-seeking behaviour may be different. Second, the sample size was small to enable comparative analyses that may have limited our statistical detection of small differences in the IPT uptake.

## 5. Conclusion

Findings from our study reveal that the NTP policy on the provision of IPT has been effectively implemented in Rwanda under the set programmatic conditions. Despite differences in methods of study, the percent IPT uptake established in this study is higher than that reported in Malawi, Timor-Leste, South Africa, South India, and Ethiopia and similar to and lower than in Gambia and Benin, respectively. Special attention should be given to child contacts more than 3 years old, child contacts who are not children of index cases, and child contacts who are children of HIV infected persons in order to identify the challenges experienced in initiating the IPT. Future interventions should find strategies to (1) fight against social stigma, especially in TB coinfected patients, and (2) eradicate the unfriendly attitude of healthcare providers towards all patients in general and TB coinfected patients in particular.

## Figures and Tables

**Table 1:** Characteristics of the index cases of the child contacts by uptake of IPT.

Characteristics	Index cases (*N* = 72) (%)	Index case whose children took IPT (*N* = 62) (%)	Index case whose children did not take IPT (*N* = 10) (%)	*p* value
*Age group*				0.716
≤35 years old	39 (54)	34 (55)	5 (50)	
>35 years old	33 (46)	28 (45)	5 (50)	
*Sex, female*	33 (46)	28 (45)	5 (50)	0.776
*Residence of the index case*				0.641
Nyarugenge	12 (17)	11 (18)	1 (10)	
Kicukiro	20 (28)	16 (26)	4 (40)	
Gasabo	40 (55)	35 (56)	5 (50)	
*Type of health facility used by index cases*				0.262
Public	51 (71)	42 (68)	9 (90)	
Faith-based	21 (29)	20 (32)	1 (10)	
*Marital Status*				0.243
Never married	11 (15.5)	8 (13)	3 (30)	
Married	50 (69)	45 (73)	5 (50)	
Separated	11 (15.5)	9 (14)	2 (20)	
*Index case Head of household*	29 (40)	25 (40)	4 (40)	0.985
*Index case tested for HIV *	61 (85)	52 (84)	9 (90)	0.617
*Result of HIV test Positive*	18/61 (30)	13/52 (25)	5/9 (56)	0.063

IPT = Isoniazid Preventive Therapy; HIV = human immunodeficiency virus.

**Table 2:** Characteristics of the household of child contacts by uptake of IPT.

Characteristics	Total (*N* = 72) (%)	Child contact started IPT (*N* = 62) (%)	Child contact did not start IPT (*N* = 10) (%)	*p* value
*Head of household*				
*Age group*				0.501
≤37 years old	38 (53)	34 (55)	4 (40)	
>37 years old	34 (47)	28 (45)	6 (60)	
*Sex, female*	18 (25)	13 (21)	5 (50)	**0.049**
*Household monthly income*				0.149
≤50.000 Rwandan Franc	47 (65)	38 (61)	9 (90)	
>50.000 Rwandan Franc	25 (35)	24 (39)	1 (10)	
*Marital Status*				0.967
Never married	7 (10)	6 (10)	1 (10)	
Married	56 (78)	48 (77)	8 (80)	
Separated	9 (12)	8 (13)	1 (10)	
*Highest education level completed *				0.625
Never attended school	9 (12)	7 (11)	2 (20)	
Primary school	43 (60)	38 (61)	5 (50)	
Secondary school and plus	20 (28)	17 (28)	3 (30)	

*Household*				
*Number of people living in the house at the time of the diagnosis of the index case*				
One person	19 (26)	16 (26)	3 (30)	0.717
Two persons or more	53 (74)	46 (74)	7 (70)	
*Parents/caregivers had knowledge of prevention of transmission of TB* ^*a*^	66 (92)	58 (94)	8 (80)	0.192
*Parents/caregivers have knowledge on the role of IPT* ^*b*^	23 (32)	22 (35)	1 (10)	0.153

IPT = isoniazid preventive therapy; TB = tuberculosis; ^a^aware about using a mask when breastfeeding, avoiding sleeping in the same room or bed with child contacts, opening windows and doors for improved ventilation, practicing hygiene while coughing. ^b^Knowledgeable about the administration of INH for 6 months to protect child contacts against TB.

**Table 3:** Characteristics of child contacts eligible for IPT by IPT uptake.

Characteristics	Under five years old child contacts (*N* = 94) (%)	Child contacts who started IPT (*N* = 84) (%)	Child contacts who did not start IPT (*N* = 10) (%)	*p* value
*Age group *				
≤3 Years	66 (70)	63 (75)	3 (30)	**0.007**
>3 Years	28 (30)	21 (25)	7 (70)	
*Sex child contact *				0.504
Female	43 (46)	37 (44)	6 (60)	
*BCG_scar*				
*Yes*	90 (96)	80 (95)	10 (100)	1.000
*Children tested for HIV *	47 (50)	39 (46)	8 (80)	0.091
*HIV test Result Positive*	2 (4)	2 (5)	0 (0.0)	0.051
*Relationship to the Index case*				
Child	70 (75)	65 (77)	5 (50)	0.060
Others	24 (25)	19 (23)	5 (50)	
*Had TB-related symptom during the screening* ^a^	23 (24)	19 (23)	4 (40)/	0.252
*Share the same bedroom with index cases *	48 (51)	42 (50)	6 (49)	1.000
*Time spend with index cases*				
≥8 hours	75 (80)	66 (79)	9 (90)	0.681

BCG = Bacilli Calmette-Guerin; IPT = isoniazid preventive therapy, TB = tuberculosis; HIV = human immunodeficiency virus; ^a^TB-related symptom = to have one of those symptoms (a cough, fever, and weight loss).

**Table 4:** Risk factors for nonuptake of IPT.

Factors	OR (95% CI)	*p* value	aOR (95% CI)	*p* value
*Child contacts*				
>3 Years	7.0 (1.65–29)	0.008	29 (2.17–400)	0.011
HIV positive	5.0 (1.0–25)	0.050	10 (0.61–174)	0.105
Not child of the index case	3.4 (0.89–13)	0.072	59 (2.74–127)	0.009
*Index cases*				
HIV-positive	4.0 (0.97–16.77)	0.054	8.1 (2.53–537)	0.038
*Household factors*				
Sex of the head of the household	4.6 (1.18–17.9)	0.028	-	
Income < 50.000 Rwandan Franc	0.1 (0.22–1.57)	0.123	0.1 (0.01–1.01)	0.050
*Heath facility factors*				
Provider not friendly	10 (1.26–83)	0.029	19 (2.514–140)	0.017

IPT = isoniazid preventive therapy; HIV = human immunodeficiency virus; CI = confidence interval; OR = odds ratio; aOR = adjusted OR.
